# Neurodegenerative disease of the brain: a survey of interdisciplinary approaches

**DOI:** 10.1098/rsif.2022.0406

**Published:** 2023-01-18

**Authors:** Franca Davenport, John Gallacher, Zoe Kourtzi, Ivan Koychev, Paul M. Matthews, Neil P. Oxtoby, Laura M. Parkes, Viola Priesemann, James B. Rowe, Stephen W. Smye, Henrik Zetterberg

**Affiliations:** ^1^ Freelance writer and King’s College London, London, UK; ^2^ Director of Dementias Platform, Department of Psychiatry, University of Oxford, Oxford, UK; ^3^ Professor of Cognitive Computational Neuroscience, Department of Psychology, University of Cambridge, UK; ^4^ Senior Clinical Researcher, Department of Psychiatry, University of Oxford, Oxford, UK; ^5^ Consultant Neuropsychiatrist, Oxford University Hospitals NHS Foundation Trust, Oxford, UK; ^6^ Department of Brain Sciences and UK Dementia Research Institute Centre, Imperial College London, Oxford, UK; ^7^ UCL Centre for Medical Image Computing and Department of Computer Science, University College London, Gower Street, London, UK; ^8^ School of Health Sciences, Faculty of Biology, Medicine and Health, The University of Manchester, Oxford Road, Manchester, M13 9PL, UK; ^9^ Geoffrey Jefferson Brain Research Centre, Manchester Academic Health Science Centre, Manchester, UK; ^10^ Max Planck Group Leader and Fellow of the Schiemann Kolleg, Max Planck Institute for Dynamics and Self-Organization and Bernstein Center for Computational Neuroscience, Göttingen, Germany; ^11^ Department of Clinical Neurosciences, MRC Cognition and Brain Sciences Unit and Cambridge University Hospitals NHS Trust, University of Cambridge, Cambridge, UK; ^12^ School of Medicine, University of Leeds, Leeds, UK; ^13^ Department of Neurodegenerative Disease, UCL Institute of Neurology, Queen Square, London, UK; ^14^ Department of Psychiatry and Neurochemistry, Institute of Neuroscience and Physiology, the Sahlgrenska Academy at the University of Gothenburg, Mölndal, Sweden; ^15^ Clinical Neurochemistry Laboratory, Sahlgrenska University Hospital, Mölndal, Sweden; ^16^ UK Dementia Research Institute at UCL, London, UK; ^17^ Hong Kong Center for Neurodegenerative Diseases, Clear Water Bay, Hong Kong, People's Republic of China

**Keywords:** neurodegenerative diseases, brain, physics, data, imaging, models

## Abstract

Neurodegenerative diseases of the brain pose a major and increasing global health challenge, with only limited progress made in developing effective therapies over the last decade. Interdisciplinary research is improving understanding of these diseases and this article reviews such approaches, with particular emphasis on tools and techniques drawn from physics, chemistry, artificial intelligence and psychology.

## 1. Introduction: the continuing challenge of neurodegenerative disease

Neurodegenerative diseases are conditions that lead to progressive injury of nerve cells, predominantly in the brain, and include Alzheimer's disease (AD), Parkinson's disease, dementia with Lewy bodies, vascular dementia, Huntington's disease, frontotemporal dementia, progressive supranuclear palsy, motor neurone disease and Creutzfeldt-Jakob disease. They are incurable and difficult to treat. Together, the dementias are responsible for the greatest societal and economic burden of all diseases in developed countries.

There are 850 000 people in the UK living with dementia, at a cost of over £26 billion per year. This is predicted to double by 2040. The scale and urgency of the problem has been recognized as a government national priority in the ‘Dementia Moonshot’ [1] and the 2021 Life Sciences Vision [2]. Despite this commitment to improving our understanding and treatment of neurodegenerative diseases of the brain, public funds for research in the area are limited and neurodegenerative diseases remain a major challenge for medical science. Over the last 20 years, billions of pounds have been invested by the private sector in clinical trials of possible treatments for dementia but it remains challenging to demonstrate clinically relevant disease modification; one recent drug, Aducanumab was approved by the FDA but rejected by the European Medicines Agency, whilst, more recently, Lecanemab has demonstrated some benefit.

There are many causes and subtypes of late life dementia. The four most common are Alzheimer's disease, vascular dementia, frontotemporal dementia and dementia with Lewy bodies. This diversity is increased with clinical and pathological heterogeneity within each disease, and complex interactions between the genetic and environmental determinants. In addition, the biochemical changes responsible for neurodegenerative disease may start in the brain decades before the clinical expression of pathology which is observed as a decline in thinking and memory. Understanding when this biochemical stage begins and what triggers it, and then distinguishing those primary causes from the cascade of secondary consequences in the brain, is an important and difficult challenge to meet.

We identify several major scientific questions in dementia research which need addressing and require interdisciplinary approaches.

First, there is a need to discriminate between different forms of dementia on the basis of underlying molecular pathology and to do this early on in the disease course, so we can ascertain a mechanistic distinction in their initial stages. Second, following on from that, there is a fundamental gap in knowledge about how brain circuits fail in neurodegenerative disease and the different processes that lead to the expression of symptoms. Third, there is a need to identify what cells are involved at what points in disease progression and to characterize the earliest stages in pathogenesis. Fourth, it is important to determine the rate-limiting steps in the spread and progression of disease, whether within or between different brain regions. There is also the challenge of developing diagnostic approaches to discriminate between the types and subtypes of neurodegenerative disease, thereby ensuring that the therapeutic interventions selected for each patient are the most appropriate. Practical and experimental challenges arise because these questions cross multiple spatial scales, from molecular through to whole-body interactions. How can we link treatable processes occurring at the scale of chemical signals or misfolded proteins that pass between different cells, through immune and metabolic interactions between brain and peripheral organs, to changes in complex human behaviours? How can we link molecular and vesicular processes at synaptic connections to macroscopic properties of whole-brain networks in promoting or responding to pathology?

Finally, there is a clear need for intermediate phenotypes, which are discoverable with advanced technologies, to be linked to clinically meaningful outcomes. There are three related challenges for developing intermediate phenotypes as clinically useful measures: the first is to establish an association between the intermediate and the clinical phenotype in the context of use. For example, to use brain hippocampal volume as an intermediate phenotype for AD-mediated neurodegeneration, a relation between hippocampal atrophy and current or future AD needed to be established [3]. The specificity of the relationship can be explored; for example, while mild-moderate brain atrophy is associated with AD, reductions in total brain volume also are associated with other neurodegenerative diseases such as vascular dementia [4] and multiple sclerosis [5]. The specificity of the association can provide information on the potential diagnostic utility of the measure.

The second challenge is to test for a causal (necessary) role of the intermediate phenotype in determining the clinical phenotype in the context of use. To extend the previous example, this would involve testing whether the development of AD in people at risk was necessarily associated with hippocampal brain volume loss [6] and, ideally, that, in the context of use, hippocampal brain volume loss always was associated with AD. This often may involve development of an understanding of the mechanistic relationship between intermediate and clinical phenotypes, e.g. between brain volume loss and neurodegeneration with AD in this case. In practice, this may involve both defining the relationship under ideal circumstances and sources of deviation from these ideal circumstances, e.g. with fluid shifts in the body [7].

A third and critical challenge is to establish a robust operational definition of the intermediate phenotype. In the case of brain volume measurements based on magnetic resonance imaging (MRI) scanning, this would be the demonstration that brain volume measurements are highly reproducible between scanners, operators and protocols for measurement [8]. In practice, despite the mechanistic clarity of brain volume loss as an intermediate phenotype for neurodegeneration, this, as well as specificity, has been difficult to establish: the measures depend on all of the factors identified. Validation of the robustness of measures is much more advanced for soluble biomarker measurements, by contrast.

The scale and nature of these challenges require a deeply interdisciplinary approach to coordinate and collate data and share mechanistic insights. Interdisciplinary research can bring tools, analytical strategies and viewpoints from different areas to potentially reframe the problem of neurodegenerative disease of the brain and offer novel solutions. For example, applying tools and techniques from statistical and soft matter physics can provide a quantitative framework to understand the underlying basic science of neurodegenerative disease and capture the multiple scales at which it works. Similarly, insights from machine learning, artificial intelligence (AI) and computational science can enable the development of scalable, practical, unobtrusive tools to stratify patients, inform clinical trials and ultimately personalize medicine.

This review aims to survey some of the most innovative and potentially impactful pieces of interdisciplinary research which draw on approaches and techniques involving the physical sciences and mathematics. It is broad in scope and based on material collated from a Rosetrees interdisciplinary workshop on neurodegenerative disease of the brain (see Acknowledgements). It starts by considering the clinical perspective in order to ensure the review focuses on questions that are important to patients and healthcare professionals. The paper then reviews novel measurements (including biofluid and digital biomarkers) and imaging before considering how to integrate and interpret these different measurements with tools from AI and machine learning, and other physics-based models.

## 2. Understanding disease mechanisms to identify better phenotypes and biomarkers

### 2.1. Cohort studies

Meeting the challenge of measuring—with the aim of modifying—the early course of neurodegenerative diseases is central to progress in the field. The analysis of large longitudinal cohort studies (table 1 for examples) may provide insight into the course of dementia and help to identify early signs of disease that are vital to understanding what happens in the period in the years before the observable clinical syndrome manifests. This in turn can inform the design of clinical trials to ensure interventions are tested in the right population, at the right time and with the right measures of success.

**Table 1.** Examples of cohorts to study neurodegenerative disease.

study	participants	no of participants	no. of years	measures	location
‘PREVENT’ (Alzheimer's disease) https://preventdementia.co.uk/for-researchers/	40–59 years old	700	5 years	cognitive assessments, MRI, genetics, biomarkers (blood and CSF in some)	UK and Ireland
EPAD (European Prevention of Alzheimer's Dementia) https://ep-ad.org/	aged over 50—no dementia diagnosis	2100	2015–2020	cognitive assessments, brain scans, samples	Europe
ADNI (Alzheimer's Disease Neuroimaging Initiative) https://adni.loni.usc.edu/	those with AD, elderly controls and those with MCI	approx. 1900	2004– (4 phases)	brain scans, genetic profiles, fluid biomarkers, neuropsychology	North America, worldwide
PiPPIN (Pick's Disease and Progressive Supranuclear Palsy Prevalence and Incidence Study) https://ftd.neurology.cam.ac.uk/PIPPIN	people with syndromes associated with frontotemporal lobe degeneration	365	2013– (3 phases)	blood test, neuropsychological tests, MRI scans	UK
GENFI (Genetic Frontotemporal Dementia Initiative) https://www.genfi.org/	people with or at risk of familial frontotemporal dementia	1100	2012–2022	cognitive, motor and oculomotor assessment, MRI, genetics, biomarkers (blood and CSF)	Europe and Canada
Insight 46 CNS sub-study of 1946 British Birth Cohort https://nshd.mrc.ac.uk/study-member-information/current-studies/neuroimaging/	study members selected at random from those who attended a clinical visit at 60–64 years	500	2015–2019	clinical, neuropsychological, β-amyloid PET and MRI, biomarker and genetic information	UK
OASIS-3 (Open Access Series of Imaging Studies) https://www.medrxiv.org/content/10.1101/2019.12.13.19014902v1	609 cognitively normal adults and 489 individuals at various stages of cognitive decline ranging in age from 42 to 95 years	1098	30 years	neuroimaging, clinical, cognitive and biomarkers	USA
AIBL (Australian Imaging Biomarkers and Lifestyle Study of Ageing) https://aibl.csiro.au/	patients with Alzheimer's disease (AD), mild cognitive impairment (MCI) and healthy volunteers	1000 + patients (minimum age 60 years)	2006–2011	MRI, PiB PET images and clinical data	Australia
DIAN (Dominantly Inherited Alzheimer's Network) https://www.alzheimers.gov/clinical-trials/dominantly-inherited-alzheimer-network-dian#description	familial AD-causing mutation carriers and their children	700	2009–2024	abnormal amyloid in the brain and spinal fluid, changes in brain size and brain metabolism	USA
PPMI (Parkinson's Progression Markers Initiative) https://www.ppmi-info.org/	4000 participants from 50 international sites at all stages of disease from prodromal to moderate and healthy participants	approximately 4000 in-person, plus more remote/online	2010 (2 phases)	clinical features, imaging outcomes, biologic and genetic markers, and digital outcomes	worldwide
‘Track-HD’ (Huntington's disease) https://portal.dementiasplatform.uk/CohortDirectory/Item?fingerPrintID=TRACK%20HD	individuals without HD but carrying the mutant HTT gene (i.e. premanifest HD), patients with early HD and healthy control individuals matched by age and sex to the combined HD	123 controls, 120 premanifest gene carriers and 123 early HD	2010–2013	3 T MRI, clinical, cognitive, quantitative motor, oculomotor and neuropsychiatric assessments	Leiden, London, Paris and Vancouver

Currently, regulators define success by clinical benefit and measures of cognitive ability. However, in the early stages of disease these may not be affected to the extent where they can be observed and measured. This silent period may only be silent because the tools currently in use are not sensitive enough to pick up changes in the brain and neuropsychological function.

It is important to note that the sensitivity and interpretation of a test depends in large part on the neurocognitive systems it requires, and the functional-anatomical specificity of a disease. The four-mountains task, for example (see below), relies heavily on hippocampal-mediated navigational representations. This makes it especially sensitive to Alzheimer's disease and its early pathology in medial temporal lobe. The test is not very sensitive to frontotemporal dementias (including TDP43 pathologies), except in severe disease where the behavioural disorder and lack of cognitive control interferes with the performance of the task as a whole (irrespective of its memory demands). Other pathologies that affect the medial temporal lobe are liable to affect performance on the four-mountains task, for example stroke of encephalitis. In that respect, the task is not specific to the molecular pathology of AD, rather the functional anatomy of the task's critical cognitive processes. Other bedside tests are preferentially sensitive to frontotemporal dementia: for example, the frontal assessment battery (FAB), the INECO screen, or Hayling test [9]. These tests require inhibition of a standard response (and sometimes the substitution by a novel answer) and depend on frontal cortical systems, which are severely impaired by frontotemporal dementia and related disorders like progressive supra-nuclear palsy.

Typically, cognitive tests lose their specificity in late stages of disease, as ‘non-specific’ deficits increasingly interfere with the performance of multiple tasks. For example, advanced dementia may affect memory, attention, language, perception and motor control, impairing performance of any task with visual stimuli and written or spoken answers over a period of several minutes. As such, neurocognitive profiles may converge with time [10], even if the molecular basis of disease is unchanged.

### 2.2. Alzheimer's disease: clinical expression versus neuropathology

When Alzheimer first described the disease that later took his name, he defined it with two key post-mortem pathologies: the amyloid plaque and the neurofibrillary tangle. In 1985, it was shown that plaques mainly consist of 42 amino acid-long amyloid beta [11]. Shortly thereafter, the main constituent of tangles was identified as hyper-phosphorylated tau protein [12]. At the time, these pathologies could not be detected *in vivo* and studies were based primarily on a clinical phenotype which relied on measures of memory and cognition.

**Figure 1.**
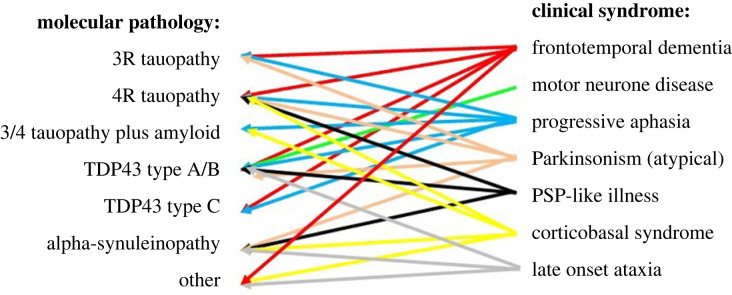
Molecular versus phenotypic precision. The classical presentations of neurodegenerative disease led to identification of their underlying molecular pathology. However, those pathologies in turn have been associated with diverse clinical phenotypes. Although some clinico-pathological correlations are strong, none are wholly exclusive. Even where autosomal dominant genetic disease gives molecular precision, pleiotropy leads to diverse clinical manifestations of the disease. (Figure prepared by Rowe.)

It is now possible to use imaging and biofluid-based biomarkers to observe the developing pathology of Alzheimer's (figure 1). Research has identified changes in the proteins amyloid and tau as defining features of Alzheimer's disease in the brain, and these can be measured using scans and cerebrospinal fluid (CSF) samples, and more recently blood samples (see §3.1).

These measurements are being collected in several longitudinal trials (table 1) and are increasingly used to support the clinical diagnosis of Alzheimer's disease. However, biological measures are still not considered the most relevant outcomes in late-phase clinical trials, with the goal to deliver clinically effective treatment, and none of them has been validated as surrogate biomarkers for clinical efficacy. This creates a dilemma for disease-modifying drugs that aim to prevent dementia by application in the pre-symptomatic phase, because clinical endpoints are the norm for gaining regulatory approval. Precise assessment of relationships between pathology and clinical efficacy is also challenging because cognitive outcomes may not map directly and consistently to biological measures of disease. For Alzheimer's disease and other dementias, there is a need to develop meaningful primary outcomes that reflect the pre-clinical biological processes, especially if there is an intention for clinical trials to be conducted with people early in disease progression (including the very long pre-symptomatic or pre-diagnostic stages of disease [13]. In the absence of outcomes which reflect pre-clinical processes, such clinical trials would have to be run for many years—perhaps decades—for clinical efficacy to be assessed.

### 2.3. New cognitive phenotypes linked to hippocampal function

Linking brain to behaviour in early stages of Alzheimer's disease requires more sensitive and reliable measures of both the neural systems at fault and the cognitive deficit. A candidate for a non-invasive biologically anchored measure of cognition has been informed by imaging innovations with 3 T and 7 T MRI that can quantify the hippocampal subfields (molecular layers within the hippocampus) that subserve particular neuropsychological functions, providing insight into the role of hippocampal place cells in spatial memory [14]. These can be associated with more sensitive cognitive tests such as the four mountains test [15], which could provide a phenotypic assay related to hippocampal degeneration.

Performance on the four mountains test correlates with the CAIDE dementia risk score (cardiovascular risk factors, ageing and incidence of dementia) in people around 50 years old with a genetic risk for dementia and early symptoms [16]. There is a strong correlation between ApoEe4 genotype and atrophy at the molecular layer of the hippocampus [17]. As hippocampal subfields have been shown to be an early area for accumulation of neurofibrillary tangles, this cognitive measure could potentially represent underlying biological processes in early disease, indicating its potential as a scalable cognitive measure that also measures changes in the hippocampal subfields. Advances in virtual reality may enable cognitive tests with increased sensitivity to early changes in hippocampus-dependent spatial memory [18].

As a disease like Alzheimer's dementia progresses, it is not only severity that increases, but also the range (extent) of neurocognitive systems involved. The deficits go beyond spatial associative learning, to include non-spatial associative learning and non-spatial perception. For example, following navigational deficits, pathology increasingly affects regions of the brain involved in language or non-spatial visual object processing, calling for tests of language and mnemonic discrimination [19]. Such phenotypic progression also occurs following non-amnestic presentations of Alzheimer's disease [20].

Interdisciplinary research is crucial to developing these biologically underpinned phenotypes through contributions to the advancement of structural and functional imaging, mass spectrometry imaging of intracellular processes and quantification of subtle neurocognitive states.

### 2.4. Trans-diagnostic approach to increase clinical phenotype precision

Advances in identifying the molecular, genetic and cellular level of pathology in neurodegenerative disease of the brain are providing important insights, but these biological processes still remain associated with a myriad of syndromes (summarized in figure 1). Genetic pleiotropy means that even a single mutation, leading to a single molecular root cause of disease, may lead to widely divergent clinical disorders [21]. While, in the reverse direction, the more common presentations of neurodegenerative disease can have very different molecular causes. For example, tau versus TDP-43-associated behavioural variant frontotemporal dementia can be clinically indistinguishable [22].

To get precision at the level of clinical phenotype and remain strongly connected with what is happening for the patient, it has been proposed that it is necessary to embrace this variance and ask what this variability in relationships between pathology and clinical expression indicates about the cascade of pathogenic processes underlying neurodegenerative disease. This requires an approach that is not governed by the diagnostic labelling (nosology) of different forms of disease in isolation, but instead uses multi-dimensional labels based on multi-variate analysis of data concerning multiple dimensions (including biochemical, pathological and clinical) of the actual disorders.

**Figure 2.**
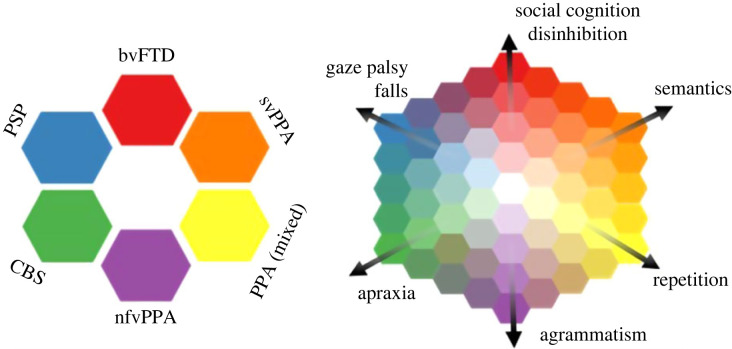
The left panel illustrates a classical approach to diagnosis, with each diagnostic group being distinct from the others, represented by a distinct colour. However, detailed characterization of clinical cohorts (for example, [10]) has shown that intermediate phenotypes are common, and syndromes are not discrete. The right panel illustrates an alternative trans-diagnostic approach, with phenotypic precision along principal dimensions of disease expression. Classical phenotypes exist, but an individual my lie at any point in a continuous ‘colour space’ of clinical phenotypes, and move across the ‘colour map’ as their disease progresses. The anatomical, neurochemical or genetic determinants of the dimensions of disease can be identified, and symptomatic treatments applied according to presence of a clinical feature rather than diagnostic label. Adapted with kind permission from figure published in Rowe [21].

Trans-diagnostic approaches set aside traditional diagnostic boundaries and focus on symptoms and mechanisms that are present across multiple disorders. For example, this approach is taken in the PIPPIN study (table 1) which looks at the relationship between the presence of one symptom and the presence of other symptoms across participants. There is clustering of signs and symptoms, into *phenotypic dimensions*, that are manifested with continuity rather than categorical differences across the population of patients. This creates a high dimensional space, on which vertices represent the classical syndromes, whereas patients can exist anywhere within the space [10] (figure 2). The dimensions, rather than the simple clinical diagnostic labels, can then be used to examine the brain structural or physiological basis of the disorders.

### 2.5. Better phenotypes for impulsivity: GABA and beta-desynchronization

This trans-diagnostic approach can yield insight into the biological mechanisms that influence important behaviours that traverse multiple diagnostic groups, and point to potential targets to treat behaviours. For example, the behaviours of apathy and impulsivity in frontotemporal dementia are features of diverse diagnostic groups and are central to the patient (and carer) experience. They are a target for improving quality of life, survival and health economic impact. By building precision into a model in terms of intermediate layers of causal mechanisms from biology to expression of this important phenotype it is possible to identify clinical trials' tools that are closer to the effect (behaviour) than the cause (biology).

Tools developed from interdisciplinary research are highly instrumental in this search for intermediary phenotypes. For example, the development of ultra-high field MRI at 7 T has increased sensitivity sufficiently to detect the reduced levels of GABA (the principal inhibitory neurotransmitter) in patients [23] and shown that levels of cortical GABA correlate with the severity of syndromes associated with frontotemporal lobe degeneration, particularly their impulsivity [10].

Magnetoencephalography (MEG) is a safe and well-tolerated means to study neurophysiology in patients with dementia, including Alzheimer's disease [24], frontotemporal dementia (FTD) and progressive supranuclear palsy (PSP). This non-invasive neurophysiological method has shown good agreement with intracranial recordings, making it suited for experimental medicine [25]. Advances in MEG have enabled the understanding of individual differences in beta-desynchronization which are important determinants of cognition and are impaired in frontal parts of the brain with FTD and PSP. Such MEG can detect the restoration of function through the use of medication that aims to increase GABA-ergic transmission [26]

### 2.6. Appling physics-inspired local cortical function models to predict efficacy of interventions in dementia

Detailed biophysically informed models of cognitive physiology are providing a way to interrogate the mechanisms behind the effects of new therapeutics for dementia. In doing so, they enable the effectiveness of a drug to be predicted, and which patients may benefit most.

The interdisciplinary approach uses detailed models of local cortical function across different cortical layers [16,27,28], known as canonical microcircuits. These enable construction of a computational representation of neuronal dynamics in brain areas of interest and a link between these local microcircuits and brain-wide networks. The approach has many forms of validation, including the accurate generation of MEG and electroencephalogram (EEG) observations and individual differences in neurochemistry [26,29].

The modelled strength of frontal-temporal connectivity correlates with clinical severity of dementia and confirms that the drug tiagabine, as expected, increases the efficacy of local inhibition. Use of MEG and application of local cortical modelling can bring precision to an intermediary level that is close to the clinical phenotype, potentially allowing predictions of how possible interventions work and for which patients. Importantly, this approach provides phenotypic and pharmacological specificity that applies across multiple diagnostic groups.

### 2.7. Imaging phenotype of locus coeruleus predicts response to drugs

Many common dementias lead to degeneration of the locus coeruleus which is the principal source of the brain's noradrenaline—a modulatory neurotransmitter that influences arousal, attention, inhibition, memory and other cognitive functions, in part by regulation of signal-to-noise in neural networks [30].

Several drugs can be used to modulate the noradrenergic system—for example, to restore behavioural control in impulsive brain disorders. There is a highly individualized response to treatment and it is important to be able to predict who will respond favourably. New neuromelanin-sensitive sequences for ultrahigh field MRI enables the detailed spatial structure of the locus coeruleus to be studied *in vivo* [31]. The integrity of this small brainstem nucleus—its contrast-to-noise—is greatly reduced in patients with several neurodegenerative diseases including Parkinson's disease and PSP [32]. Using ultra-high field MRI, it is possible to predict how a patient will respond to noradrenergic drugs according to the locus coeruleus contrast-to-noise ratio [33].

This is another example of how advances in technology are allowing increased precision in characterizing phenotypes and, through the modelling of intermediate phenotypes with insights from physics, it is possible to make a clearer link from root causes to symptoms and novel treatments.

## 3. Improving accuracy, accessibility and continuity of biomarkers

### 3.1. Current state of fluid biomarkers for neurodegenerative disease

Biomarkers had been used to classify patients with Alzheimer's disease for over a decade when it was proposed as a means to standardize definition and differentiate true Alzheimer's disease from neurocognitive disorders that do not display Alzheimer's disease pathology. This became known as the A/T/N system (table 2). However, there are still discussions as to what exactly the different biomarkers represent. For example, it is generally understood that tau is not a direct biomarker for neurofibrillary tangles but a biomarker for the neural response to amyloid [35]. The state of biomarkers for neurodegenerative disease of the brain is continually progressing and interdisciplinary research is playing an important role.

**Table 2.** General description of the most important measurement technologies for Alzheimer's disease biofluid-based biomarkers [34]. The table includes technologies that may be useful for both blood- and CSF-based biomarkers but some of the biomarkers are present at very low concentrations in blood, which may require ultrasensitive assays (more sensitive than ELISA).

technology	explanation
sandwich enzyme-linked immunosorbent assay (ELISA)	The target analyte is captured between two antibodies (capture and detection). The capture antibody is immobilized onto a surface (often the plastic surface of a well, e.g. in a 96-well plate). The detection antibody is labelled with an enzyme that produces a measurable signal (fluorescence or colour) by converting a substrate to a product. The lower limit of quantification of an ELISA depends on the antibodies and the target analyte but is often in the nano- to pico-molar range.
immunoassay with electrochemiluminescence detection (ECL)	A variant of ELISA but instead of an enzyme, the detection antibody is labelled with a molecule that directly produces luminescence during an electrochemical reaction. This detection principle is often a little bit more sensitive than ELISA.
single molecule array (Simoa)	This is a classical sandwich ELISA, but the capture antibody is conjugated to magnetic beads instead of the bottom of a 96-well plate, and the sandwich complexes (bead, capture antibody, target analyte and enzyme-labelled detection antibody) are pulled down in microwells (one bead per well), where the detection reaction is allowed to occur. This compartmentalized detection reaction in a very small volume allows for the detection of the biomarker at the single molecule level. In biofluids, the Simoa assays can be 100 to 1000 times as sensitive as a regular ELISA (sub-femtomolar analytical sensitivity).

**Figure 3.**
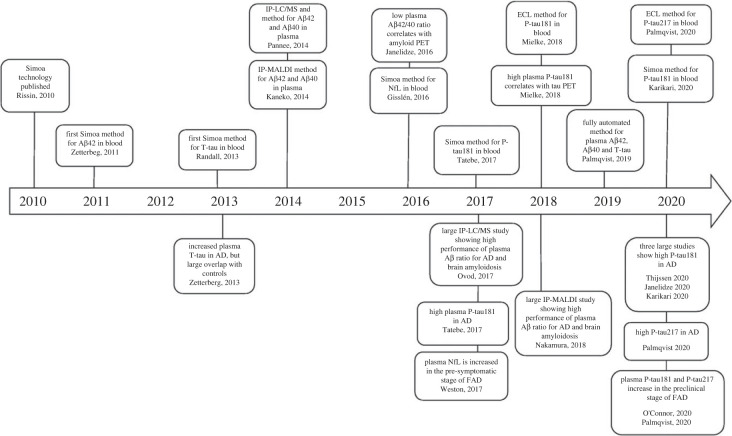
Timeline for blood biomarker developments during the last decade (figure reproduced from from [34] by kind permission of the publishers under Creative Commons licence https://creativecommons.org/licenses/by/4.0/). Abbreviations: Simoa, single molecule array; A*β*42, the 42 amino acid form of amyloid *β*; T-tau, total-tau; IP-LC/MS, immunoprecipitation liquid chromatography-mass spectrometry; IP-MALDI, immunoprecipitation matrix-assisted laser desorption/ionization; A*β*40, the 40 amino acid form of amyloid *β*; NfL, neurofilament light; P-tau181, tau phosphorylated at amino acid 181; P-tau217, tau phosphorylated at amino acid 217; ECL, electrochemiluminescence [34].

Traditionally, fluid biomarkers for Alzheimer's have been measured in cerebrospinal fluid (CSF), but technological progress has resulted in improved analytical sensitivity, making it possible to measure biomarkers in standard blood samples (figure 3), which is much more amenable to patients and research participants and therefore enables more powerful data. There are continuing challenges to blood biomarkers in that the concentrations of the relevant CNS amyloid signal are lower in blood than in CSF as there is also a backdrop of non-relevant amyloid derived from blood platelets, hepatocytes and muscle. New techniques are helping overcome these.

These techniques include immuno-precipitation, which uses antibodies to extract amyloid from the blood combined with magnetic beads to measure the 40/42 ratio by mass spectrometry (table 2). Measurement of P-tau in blood has been enabled by modifying commercially available single molecular array (Simoa) assays using antibodies previously employed in enzyme-linked immunosorbent assays for CSF testing, as well as through the generation of novel immunoassays, and mass spectrometry-based methods are showing encouraging results in comparison with positron emission tomography (PET) measurements in applications with a range of different patient groups. For example measurements of P-tau in the blood can identify those with familial Alzheimer's disease, those with pre-clinical amyloid, those individuals with Down's syndrome who start to develop Alzheimer's disease, and distinguish between Alzheimer's and other neurodegenerative diseases such as frontotemporal dementia and Parkinsonian disorders [36–38].

There is also progress on neuro-filament light as a blood-based biomarker for neurodegeneration, which can be measured using Simoa assays and mass spectrometry. This allows identification of neurodegenerative diseases in general, including early onset genetic forms of Alzheimer's [39] and Down's syndrome dementia [40]. With the increasingly recognized value of longitudinal, population-based cohort studies in studying disease progression over time, these blood-based biomarkers offer a valuable way to integrate fluid biomarkers into data collection initiatives such as those of the UK Biobank.

### 3.2. Continuing role of interdisciplinary research in development of fluid biomarkers

On the horizon there is a need to continue work on other biomarkers that may reveal more insight into pathology, e.g. CSF biomarkers for lyosomal dysfunction, blood–brain barrier dysfunction, astrocyte activation, microglial activation and alpha-synuclein pathology [41]. Further work is also needed on the elusive TDP-43 pathology that underlies dementia and cognitive decline but for which imaging or fluid biomarkers have not been developed. Recent work shows promise in the early detection of seeds of misfolded TDP- 43 [42].

Interdisciplinary research is key to develop and improve techniques to measure and assess fluid biomarkers, and to establish their use in clinical work through comparing them with MRI, PET, genetics and clinical phenotypes. Work on the development of clinical chemistry tests on fully automated instruments could produce results in Alzheimer's disease biomarkers in less than 20 min that are both of high precision and amenable to high throughput [43]. This bodes well for full implementation of these biomarkers in clinical laboratory practice with uniform reference limits that are used globally.

In many European countries, CSF biomarkers are already in use in clinical laboratory practice in accordance with country-specific regulations. Similar work is now happening for the new blood biomarkers. For example, plasma neurofilament light chain (NfL) is available as a test in clinical laboratory practice in Sweden, The Netherlands and France, and plasma A*β*42/A*β*40 ratio is clinically available from a lab in St. Louis, MO. If this is proving successful, interdisciplinary research will be necessary to establish the requirement for biomarkers to translate more widely into the clinic, to combine their measurement with other data, and to develop appropriate use criteria and interpretation guidelines (see §4.3)

### 3.3. Digital technologies for continual and widespread pre-clinical detection

The field of digital health has seen significant progress both in terms of its technological development and its implementation in healthcare settings. It has a particular value for fields like neurodegenerative disease where researchers are aiming to capture data before the clinical syndrome is observable and in large samples. In this context, digital technologies offer a scalable, practical and unobtrusive solution to monitoring and enabling better reach of research with implications for democratization and inclusivity.

Research is showing that the rapid escalation in tau protein and decline in amyloid indicates when individuals enter the more aggressive phase of Alzheimer's disease [44] and provides a potential opportunity for medical treatments to have the most benefit. However, a major challenge is in trying to identify this phase before it happens in pre-clinical cognitively healthy people where the amyloid level is often not detectable. Digital technologies allow remote, continuous and affordable monitoring in a participant's own environment, potentially detecting small changes in cognition before clinical symptoms are identified; this may be particularly valuable in people known to be at high risk of dementia [45].

There are two main approaches to capturing data: active and passive cognitive monitoring. In active monitoring, cognitive tests are completed using remote devices, which endow particular benefits in terms of assessment at multiple times. Implemented in the PREVENT study (table 1), analysis of active cognitive monitoring has demonstrated the ability to detect accelerated memory extinction as an early indicator of cognitive decline risk [46]. Assessment of engagement with the app showed a promising geographical spread across the UK. Tests are also using virtual reality to enable people to hide and find a virtual object, providing insightful data on the likelihood of later cognitive decline [47].

### 3.4. Harnessing digital developments in active and passive cognitive monitoring

Passive cognitive monitoring can include speech monitoring and the use of modified browsers to assess the speed of typing and reading or to measure performance in interactions with smartphones such as finding names or spelling or outdoor navigation using the global positioning system (GPS). There are also developments in the use of big data approaches on people's Internet searches and social media engagement related to neurodegenerative disease. Wearable devices and Bluetooth beacons are being used to record signals to triangulate and monitor movement to assess navigation and speed as measures of spatial performance. Wrist-worn actigraphy is a relatively mature method that can be used to evaluate levels of physical activity and sleep quality, both consistently linked with dementia risk [48,49]. Work is ongoing to optimize wearable technology to extract reliable variables.

The field of active cognitive monitoring is also developing quickly and witnessing more collaborations between software developers and academia allowing apps to be rooted in good science for more meaningful data for clinical practice. There are many interdisciplinary research opportunities in this area, particularly using approaches from physics, to work on the densely sampled time series that are emerging from these projects and the application of machine learning to reduce data complexity, which is typically high—for example, virtual reality data approaches can yield 700 individual variables (table 3).

**Table 3.** Models to implement and use digital data.

type	description	examples
volunteer Registry	large scale and consent to be re-contacted	Join Dementia Research (https://joindementiaresearch.nihr.ac.uk)
stratified recruitment registry	explicit consent for re-contact on the basis of dementia risk in cognitively healthy volunteers	Dementias Platform UK Clinical Studies and Great Minds Register [50]
trial ready cohort	explicit consent to re-contact and at-risk groups with extensive phenotyping	EPAD (see table 1)

Looking to the future, there is a need for data linkage work especially when using clinical data alongside better data mining techniques to extract insight from digital interaction data and make it accessible and valuable (see §4). Further work is also needed on risk stratification algorithms to include these variables into some form of prediction model that can also include data from fluid biomarkers and genotypes, and potentially polygenic risk scores. All these will require interdisciplinary working and potential insight from statistical physics and AI. Early examples of multi-modal, cross-cohort data integration resources dedicated to accelerating dementia research are Dementias Platform UK (https://www.dementiasplatform.uk/) [51] and the Alzheimer's Disease Workbench (https://www.alzheimersdata.org/ad-workbench).

It should be noted that the advances in the capability of digital technology to capture data relevant to Alzheimer's disease risk needs to be matched by analytical methods that can limit resource-intensive investigations and treatments to those that need them. Machine learning methods can be used for this purpose and in a recent analysis on routinely collected cohort data, this was shown to outperform logistic regression in the identification of both Alzheimer's and non-Alzheimer's disease pathology [52]. This approach has particular merit in low socio-economic settings and is likely to be an important element of the broader drive to address the challenge of achieving equity in the diagnosis and treatment of neurodegenerative disease of the brain.

## 4. Imaging for neurodegenerative disease and role of interdisciplinary research

There are multiple types of measurements derived from imaging that can inform research on brain structure, brain function and brain chemistry. Methods include functional magnetic resonance imaging (MRI) and positron emission tomography (PET) alongside the more neurophysiological techniques from electroencephalogram (EEG) and magnetoencephalography (MEG) that can give temporally precise measurements of brain activity. There are numerous review articles on the development and use of imaging biomarkers in neurodegenerative disease of the brain—see, for example, [53]—and this review is not intended to provide a comprehensive overview of all such imaging techniques.

Each method yields a variety of different quantitative metrics reflecting different aspects of brain anatomy, chemistry and function. These have been powerful in research settings and generated important insight into disease progression, outcomes for clinical trials (see §1) and ways to predict and stratify patients (see §5). However, similar to fluid biomarkers, they may have more potential in clinical applications than is currently in place and interdisciplinary research could be key to enabling this to happen. Furthermore, there is an active MRI physics community continually developing novel measurements sensitive to new aspects of physiology, along with chemists and biologists developing new PET tracers.

### 4.1. Challenges and opportunities of using imaging methods in context of neurodegenerative disease

Imaging can provide valuable insight into processes of disease progression, particularly in combination with biophysical models, such as those that describe the propagation of activity in brain networks and provide a quantitative framework to understand measurements (see §6.1). This mechanistic insight can inform the design of interventions to target the disease process. Imaging biomarkers may also provide important intermediate outcome measures in clinical trials that are closer to the underlying pathological processes than cognitive measures (see §1.1), potentially providing earlier and more sensitive markers of the efficacy of an intervention. If intermediate outcomes become accepted as primary outcomes, then this may allow trials to be quicker and smaller, enabling more rapid progress.

Imaging also plays a role in prediction and stratification to inform decisions about patients—for example, stratifying patients into subgroups that could benefit the most from an intervention and predicting those who will go on to develop chronic dementia. Imaging combined with machine learning is now being used to perform these types of classification (see §5).

The value of imaging in prediction has been known for some time. For example, it has been shown that pooling multi-modal imaging data with CSF measures has greater predictive power in assessing whether an individual attending a memory clinic would be diagnosed with Alzheimer's two years later than using the CSF measures alone [54]. Looking to the future, the use of data from studies such as the European Prevention of Alzheimer's Disease (EPAD) (table 1) can improve the understanding of what combinations of measures can accurately predict the future course of disease. Key to this is the ability to quantify the success of these predictors for the asymptomatic individual patient and studying how to combine different measures optimally.

### 4.2. Scanning data from at-risk groups

Scanning data from at-risk groups is valuable in the identification of functional differences, for example fMRI research has shown those who carry the APOE *ε*4 allele but who are asymptomatic already show observable differences in brain activity in the hippocampus [55] (see §2.3).

The UK Biobank has provided a step-change for this type of research through its inclusion of brain, body and heart MRI scanning in its data collection for 100 000 individuals, which allows eventual retrospective insight from the data of those participants who go on to develop neurodegenerative disease. By analysing scanning data collected before disease onset it is possible to identify early markers. Even from the data of the first 1000 UK Biobank participants (1% of the projected cohort size) researchers have developed a powerful protocol for collecting multi-modal measures, which can be acquired rapidly in 34 min, demonstrating the statistical benefits conferred by large numbers [56].

The objective is to take the rich multi-modal brain imaging dataset in combination with information on lifestyle, genetics and environment, and then use AI and machine learning to identify which combination of these measures can predict long-term health outcomes (see §5).

### 4.3. Overcoming barriers to translation from research to clinical practice

Researchers and clinicians currently work with brain scans in very different ways. Researchers consider scans as collections of data fed in a quantitative image ‘matrix’ while clinicians typically view scans as pictures illustrating macroscopic features and signs to inform a diagnosis.

Work is being done to develop routine image analysis pipelines that can produce imaging-derived phenotypes where the numbers extracted from the brain scans also reflect clinically meaningful entities. For example, the work that has been done on hippocampal subfields (see §1.2) where quantitative readouts of healthcare-based MRI scans are providing meaningful insight into a particular brain feature and its clinical relevance. These phenotypes could potentially be generated at the time of scanning but would then need to be compared with relevant population norms to be translated into clinically meaningful information. Work is being done with UK Biobank data on solutions to derive population norms that resemble growth curves (FD3) so an individual's brain scan measures can be considered in relation to those of the population.

To accelerate translation, closer integration is needed between clinical services and the research community. ‘Brain Health Clinics' are increasingly common—one example of this is in Oxford (https://oxfordhealthbrc.nihr.ac.uk/our-work/brain-health-centre/), where patients are referred for assessment, including a brain scan using research-level scans that have been optimized to provide standard clinical information and harmonized to UK Biobank data to generate population norms.

Interdisciplinary research has an important role to play in overcoming translational barriers by providing techniques and approaches for standardization and harmonization that can enable the development for robust, accurate and clinically feasible methods for quantification of image-driven phenotypes

### 4.4. Scanning for microvasculature dysfunction as a valuable avenue into neurodegenerative disease research

Clinical work around neurodegenerative research is often informed by the details of vascular lesions in the brain, such as infarcts and white matter hyper-intensities. Advances in MRI technology are now enabling better and more detailed measurements of neurophysiology of the normal-appearing tissue that can help capture dysfunction of the small blood vessels in the brain to better characterize neurodegenerative disease [57]. Vascular changes are relevant to all types of dementia, not just vascular dementia. For example, hypo-perfusion occurs early in Alzheimer's disease and the disease processes may interact mechanistically, exacerbating the progression of both [58].

There is now a wealth of evidence that dysfunctional vasculature is a contributing factor in dementia and the eponymous ‘Jack model’ [59] of disease progression has been updated to include an early vascular component [60]. Importantly vasculature's role in disease appears to happen in the period just preceding cognitive impairment, therefore imaging of the microvasculature, allows the study of biological processes in the human brain that may trigger the onset of cognitive decline and potentially provide biomarkers that could be used to test new therapeutics.

Physicists and biologists are working together to develop biophysical models on which to base new measures that can be detected by MRI scans and then validating these measures in animal models. Some of these measures are listed in table 4.

**Table 4.** Measures of microvascular dysfunction.

brain feature	measurement	reference
blood–brain barrier dysfunction	MRI of water exchange rate across the blood-brain barrier; dynamic contrast-enhanced MRI of gadolinium-based contrast agent leakage—improvements to sensitivity.	[61–63]
cerebral blood flow	arterial spin labelling MRI to measure cerebral blood flow and arterial transit time (time it takes for blood to reach the capillary bed)	[64,65]
tortuosity (or twistiness) of vessels	capillary segment length using diffusion-weighted MRI—validation against micro CT	[66]
brain oxygenation	quantitative susceptibility mapping MRI to estimate concentration of deoxyhaemoglobin in the veins and associated oxygen extraction fraction	[67–69]

In order to assess the value of microvasculature measures, a study has used simultaneous PET-MR in 13 people with mild cognitive impairment and 16 age-matched controls to assess the sensitivity of these new vascular measurements to cognitive ability and vascular disease risk and to assess their repeatability.

Analysis indicates that measures of arterial transit time and brain oxygenation appear to be both repeatable and sensitive to cognitive impairment and vascular disease risk [70]. Further work is needed, but a combination of these measures of microvasculature dysfunction using precision scanning and biophysics models could ultimately become a tool to detect and study disease early on, prior to dementia onset.

## 5. Systems for collecting and holding multi-modal data

The increasing quantity, quality and variety of data provide an exciting landscape in which to build interdisciplinary approaches to neurodegenerative disease. Within this landscape, flexible and secure informatics infrastructures enable smart and safe integration of data from multiple sources, including linkage to electronic health records.

**Figure 4.**
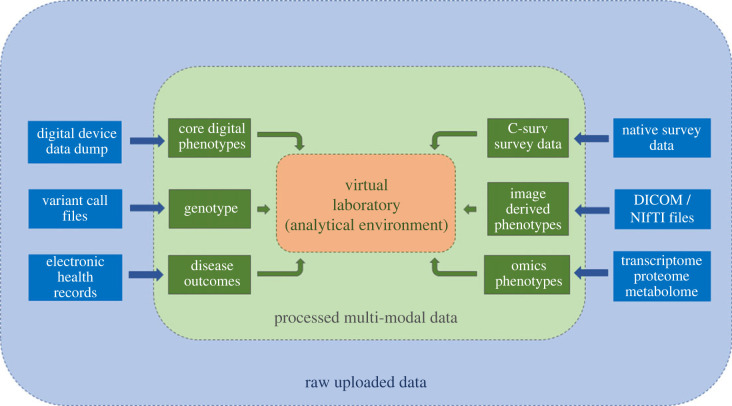
Multi-modal data analysis pipelines (DPUK Spring Academy 2022).

The challenge of bringing these data into one place to make them accessible, usable and secure is being tackled by the Dementias Platform UK (DPUK) (Welcome—DPUK, dementiasplatform.uk). The DPUK Data Portal Home—DPUK Data Portal (dementiasplatform.uk) brings multiple data assets into a single virtual location for curation, access and analysis. On the ground, this involves pre-processing raw data to make them research-ready (typically by applying a common data model), brokering access with data controllers using standard governance solutions, and providing a suite of tools for data discovery and analysis. Within the platform, there are different pipelines for each data modality including genomics, digital devices, imaging, research surveys and electronic health records (figure 4). For example, survey data using diverse data models are curated to the C-Surv data model [71] to enable rapid discovery and analysis. For genetics, specialist variable call format data are converted to genotype and polygenic risk scores, as these data are more accessible to non-geneticists. Although some of these pipelines are in development, projects or pilot-projects are running in each of them.

This approach offers a generic solution for high trust systems that are transparent, secure and fully auditable. The aim of these systems is to support interdisciplinary research by enabling specialist and non-specialist scientists to access multiple multi-modal data assets. The approach is being adopted by several international counterparts (https://www.dementiasplatform.com.au; Alzheimer's Disease AD Workbench (ADWB)—ADDI (alzheimersdata.org)).

As more data are integrated into the DPUK Data Portal and other resources in this field develop (UK Biobank, Genomics England and the UK data archive), the overarching model of data management becomes a national network of interoperable, trusted research environments, accessible through a single login, maximizing opportunities for interdisciplinary research.

## 6. Moving from data to projections with statistical physics, artificial intelligence, machine learning and computational approaches

The value that statistical physics brings to understanding collective neuronal behaviour is becoming increasingly recognized. As a discipline that is used to modelling billions of atoms, it can offer much in terms of information theory but also in terms of models of phase transitions, pattern formation and collective dynamics.

There are fundamental challenges to bridging statistical physics and neuroscience, particularly as neuronal interactions are unlike the traditional interactions studied in physics. Neuronal interactions are pulse-like, directional, time delayed and plastic, which means the classical laws of physics are not applicable. In addition, the highly dimensional topology of the brain networks makes it difficult to characterize collective properties. However, interdisciplinary research is finding ways to overcome these challenges and to enable physics to bring important insight into neuroscience and neurodegenerative diseases.

Alongside this insight from pure statistical physics, AI, machine learning and computational approaches are making huge advances in enabling predictive modelling from multi-modal data and bringing together data-driven approaches and clinical observation.

### 6.1. Using statistical physics to understand information flow and stabilities in cortical brain network

By moving between statistical physics models and data from neuronal recordings from mammals, it has become possible to quantify the spreading dynamics of neural activity in the neocortex using the mathematical framework of branching processes [72–75]. Experimental results suggest that both information transfer and task performance depend on the relations between external input strength and recurrent activity within the network [76].

Through estimating the branching parameter, which represents how activity propagates through neural networks, networks appear to work in a regime that is close to—but not precisely at—a critical point at which maximum information transfer would occur [75,77,78]. For many cortical areas, from visual cortex in cat to temporal lobe in humans, we find that cortical dynamics do not precisely operate at the critical point but about 2% away from it. Across all species, a branching parameter of 0.98 provides a match to the recorded data [72,75]. By working in this near-critical regime, the network may not transfer the maximal amount of information at all times. However, in that regime, small changes in the effective excitatory synaptic strength can strongly change the computational properties of the network [78]. This sensitivity of the network enables a fine-tuning of computational properties to different tasks [79].

The model used to describe neural network activity in terms of a branching process is relatively simplistic [80]. Nonetheless, the neural activity generated by the model is very similar to that of cortical spike recordings as measured by higher-order statistical measures; both the model and the *in vivo* activity show similar higher-order statistics, e.g. in the cross-correlation, power-spectral density and avalanche size distribution (technical definitions of the higher-order statistics given in reference [80]).

This suggests that the branching process, despite its simplicity, captures the basic propagation of spiking activity in the mammalian cortex. The core reason could be that this type of model captures the first-order approximation to the dynamics, thus the linear part of the activity propagation. And apparently that linear part of the propagation of activity seems to account for a large fraction of the network dynamics [79,80].

**Figure 5.**
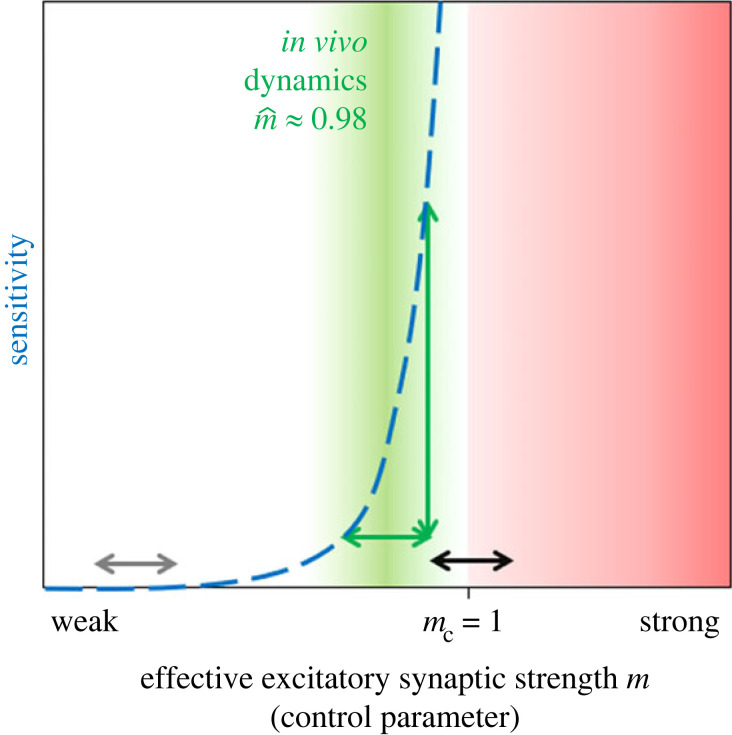
Network properties, like sensitivity to input (blue dashed line), diverge in the vicinity of a critical point (transition at *m = m_c_ = 1*). Recordings of various mammals suggest consistently that cortical areas operate in a regime close to a critical transition (green area), with a safety margin to supercriticality (red), where activity may become instable (‘bursty’ or highly correlated). In the regime close to criticality (green), small changes in the control parameter *m* lead to major changes of the sensitivity (green arrows). Thereby, computational properties like the sensitivity can be tuned to task performance. The same amount of change in *m* hardly incurs any change when the network is far from the transition (grey arrow), but risks tipping over to instability if the network is too close to the transition (black arrow). Hence, in the vicinity of the critical point, the network may profit from sensitive tuning of its network properties to task performance without tipping over to instability. Figure prepared by Priesemann.

The model together with the analysis of the diverse recordings predict that, within cortical areas such as visual cortex, prefrontal cortex and hippocampus, there is clearly more internal recurrent propagation of activity than external activation from other brain areas or stimuli [80] (figure 5). In fact, only a few per cent of the activity in a cortical area seems to originate directly from input. The vast majority is internally generated [79,81]. That is indeed in line with experimental probing of the impact of subcortical input relative to ongoing activity [82]. When thinking about dysfunction that occurs within these higher functional areas, this theoretical framework suggests that already small changes or mis-tuning in the effective excitatory synaptic strength can have a major impact on the local sensitivity of the cortical network, and hence on its information-processing abilities [72,75].

In terms of information processing, a popular hypothesis suggests that the cortex should operate precisely at the transition between stable and unstable dynamics [83]. At that ‘critical point’, a number of computational properties are maximized, such as the sensitivity or the correlation length. However, maximizing these properties does not necessarily optimize task performance in general. Interestingly, analyses of task performance on a neuromorphic chip showed that solving the more complex version of a task profits from tuning the network to the critical points. Surprisingly, however, for the simple version of the task, optimal performance was achieved when the network was tuned to a working point farther away from that critical transition [76]. This is straightforward to understand if for solving the simple version of the task the network only required integrating information over a short time window. In that case, a longer memory timescale in the network might corroborate performance. Hence, the classical interpretation that criticality would optimize task performance has to be refined—it is the complex tasks that profit from maximized computational properties, while more simple tasks may suffer from the variability [76,84]. Conceptually, these results might provide insight onto how cognitive and memory tasks enrol different types of operating points or propagation regimes. If one follows that thought, it can help to understand how this recurrent activity perhaps ‘goes awry’ in neurodegenerative disease when the fine-tuning of the network's sensitivity and working point is hampered. Combining this insight with the more detailed scanning data (see §4 above) could provide promising avenues for research.

### 6.2. Machine learning on multi-modal data to provide predictive insight

As technology and science advance, researchers are presented with the welcome challenge of an increasing amount and variety of data on neurodegeneration across timescales. Traditional analyses within disciplines remain valuable, but approaches to combine this data from different sources through AI and machine learning could potentially allow this data to be applied to clinical settings and trials.

Machine learning can help by enabling early prediction while imposing the least invasive and costly tests. By distinguishing patients that remain in a stable phase of cognitive impairment from those where the condition worsens, machine learning can also help overcome the issue of misdiagnosis or ‘misclassification’. Clinical misdiagnosis is common as it is often qualitative, or semi-quantitative at best, often relying on arbitrary cut-off points. Machine learning can provide data-driven decision thresholds that are optimized to minimize misdiagnosis rates. Additionally, machine learning systems can fuse multi-modal high-dimensional data, a process that is beyond human capabilities. This includes the possibility of assessing confounders such as comorbidities. Overall, machine learning holds out the promise of more precision, transparency and clarity, thereby enabling a truly predictive medicine.

### 6.3. Three-phase framework to model prediction with machine learning

There is a three-phase machine learning approach that is proving valuable to overcoming misclassification. The first phase extracts features that are highly predictive of changes in cognition, while the second uses a machine learning algorithm to make reliable and robust predictions. The third phase involves moving away from binary classifications to a trajectory modelling approach to reduce the risk of misclassification.

**Figure 6.**
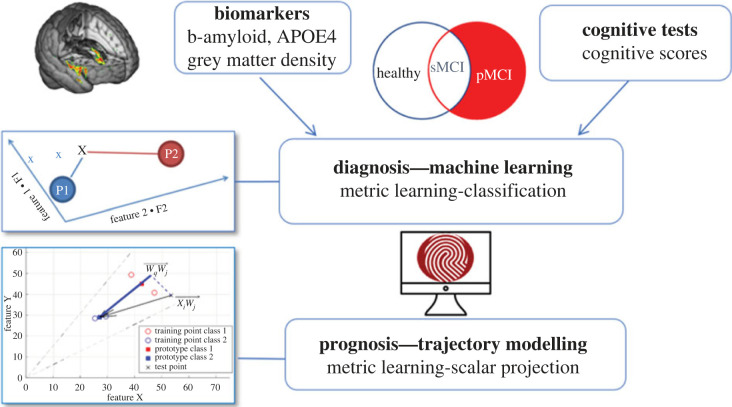
Predictive prognostic modelling approach involving feature extraction, metric learning classification (sMCI: stable mild cognitive impairment. pMCI: progressive mild cognitive impairment) and trajectory modelling. Figure prepared by Kourtzi.

Using this approach, an interdisciplinary research team from Cambridge is using baseline structural imaging data from about 500 individuals from the ADNI dataset (table 1) to derive a grey matter density score which is predictive of changes in memory. The grey matter score in combination with beta-amyloid and APOE *ε*4 was used to build the machine learning model (phase 2) which was then proven to reliably predict change in cognition (that is, memory scores) in an independent dataset (figure 6). Another test of its reliability was its ability to distinguish two classes of people based on biological markers: beta-amyloid and APOE4.

To move away from the binary classification (phase 3) the group have used a trajectory modelling approach which creates a prototype for stable health and then derives distances from this prototype for every individual, not only in terms of how far away people are from a stable prototype but also the rate of decline. For this trajectory modelling approach, biological data provide more sensitivity than cognitive data, while for the class-based discriminations the performance is similar for both types of data. This trajectory modelling approach is validated by inputting data on a separate group from the ADNI dataset and showing the model accounts for 46% of the variance in rate of memory decline [84]. The model was also able to predict accumulation of regional levels of tau in the brain over time [85].

This approach can be used to stratify patients into different groups which could be useful in clinical trials or interventions working on specific pathways. For clinical trials this approach could potentially reduce sample size by nearly a third [85]. The plans for the future are to use these approaches to build AI-enabled clinical decision support systems that help clinicians assign the right patients at the right time to the right diagnostic pathway. This in turn will improve patient well-being by reducing the requirement for invasive and expensive diagnostic tests and by guiding more precise selection for clinical trials.

### 6.4. Top-down computational models for understanding disease progression

The increasing number of studies that are collecting longitudinal multi-modal data, including MRI and PET images, genetics, cognitive tests, CSF and blood biomarkers are enabling researchers to unleash the power of computer science to unravel the complexities of neurodegenerative diseases. These data-driven approaches work alongside neurological and biological domain knowledge to enable researchers to construct models capable of simultaneously estimating the pathophysiological cascade of disease and the time axis of this trajectory, as well as subtypes thereof. Some of these statistical models are inspired by physics while others directly use the tools of physics.

One of the earliest data-driven approaches to modelling neurodegenerative disease progression was the event-based model [86]. This learns a discrete sequence, and uncertainty in the sequence, of cumulative abnormality—from only cross-sectional data. An early application of the event-based model fused multi-modal data from CSF markers, atrophy, cognition and brain volume changes into a uniquely fine-grained patient staging system that also predicted the conversion from mild cognitive impairment to Alzheimer's disease [87]. Updated versions expand the models' applicability to include the personalized discriminative event-based model [88] and the kernel density estimation event-based model [89], with applications including typical and atypical Alzheimer's disease [86,88,89], multiple sclerosis [90], Huntington's disease [91], Parkinson's disease [92] and frontotemporal dementia [93]. The subtype and stage inference (SuStaIn) algorithm [94] combines unsupervised learning with pseudo-time disease progression modelling to automatically estimate disease progression subtypes, with early applications unravelling heterogeneity in Alzheimer's, frontotemporal dementia [94,95] and predicting clinical trial treatment response in multiple sclerosis [96].

There are also continuous-time versions of data-driven disease progression models. These exploit the power of Gaussian processes and other self-modelling regression approaches [97–100] to simultaneously estimate a disease trajectory and latent disease-time score that correlate with cognitive status and are predictive of future decline in Alzheimer's [101].

### 6.5. Bottom-up models of brain connectivity-based pathology spreading to generate insight into disease mechanisms

Neurodegenerative diseases are increasingly seen as network disorders where brain connectivity plays an important role [102]. Connectivity models in the brain such as a network diffusion model [103] and epidemic spreading model [104] aim to predict the spread of pathogenic proteins and downstream pathology that is measurable (from the top, down) to provide insight into the underlying mechanisms (bottom-up) of neurodegenerative diseases.

Physics has a long history of using multi-scale modelling to understand complex systems. Converging evidence suggests that we currently have an opportunity (if not an obligation) to develop such approaches in our attempts to unravel the mysteries of neurodegenerative diseases. In short, to marry top-down models of disease phenomena, with bottom-up models of disease mechanisms, such as brain connectivity-based models. This includes leveraging machine learning and AI to improve predictions of where and when pathology will occur, which can also be informed by selective vulnerability of brain regions, and biologically informed microscale models of protein misfolding [90,105,106]. It is clear that techniques from physics and mathematics play a key role in linking the bottom-up mechanistic models to the top-down phenomenological models [107] guided by domain knowledge.

## 7. Conclusion

This review provides a range of examples of how interdisciplinary research, particularly approaches from the physical and mathematical sciences, are contributing to understanding neurodegenerative disease of the brain. Developments in technology are also improving the capacity to collect more precise data and develop novel fluid, scanning and digital biomarkers. New platforms and modelling techniques are enabling the analysis of complex and multi-modal data, and these analyses are enabling a more informed approach to the design and conduct of clinical trials, which are based on a deeper understanding of underlying mechanisms.

A greater emphasis on interdisciplinary approaches is enabling some novel reframing of problems which, perhaps, in the past became rather polarized, such as phenotypic versus mechanistic measures and top-down versus bottom-up approaches. Of course, none of this is an entirely new way of working but rather an increased enthusiasm for the iterative gathering and testing of insights from a range of different disciplines, which may lead to a better understanding of underlying disease mechanisms.

As with all scientific endeavour, the development of medical science inevitably takes twists and turns—and occasional dead ends—on its journey to develop new understanding of disease. Interdisciplinary research is integral to negotiating this journey and entails overcoming barriers that may be preventing research from gaining useful and different perspectives. Now is a valuable time to take note of the range and impact of interdisciplinary approaches that have already contributed to the field, and consider how to foster those that are emerging, in order to ensure that we harness their strengths. In so doing, we will address the ‘Grand Challenge’ of neurodegenerative diseases of the brain in a manner that is both scientifically rewarding and most likely to deliver real clinical impact.

## Data Availability

Details of how to access the data referred to in this Review are given in the text and accompanying references.
